# “No-one’s contribution is more valid than another’s”: Committing to inclusive democratic methodologies

**DOI:** 10.1177/00345237241249376

**Published:** 2024-05-10

**Authors:** Kirsty Liddiard, Louise Atkinson, Katy Evans, Barry Gibson, Dan Goodley, Jamie Hale, Rod Lawson, Katherine Runswick-Cole, Ruth Spurr, Emma Vogelmann, Lucy Watts, Kate Weiner, Sally Whitney-Mitchell

**Affiliations:** School of Education and iHuman, 7315 University of Sheffield, Sheffield, UK; Artist; Independent Researcher; School of Dentistry, 7315 University of Sheffield, Sheffield, UK; School of Education and iHuman, 7315 University of Sheffield, Sheffield, UK; Artist; Sheffield Teaching Hospitals; School of Education and iHuman, 7315 University of Sheffield, Sheffield, UK; Independent Researcher; Independent Researcher; Independent Researcher; Sociological Studies, 7315 University of Sheffield, Sheffield, UK; Independent Researcher

**Keywords:** Disability, breath, accessibility, coproduction, illness

## Abstract

In this article, we explore the power and potential of democratic research methodologies in and beyond Critical Disability Studies research contexts. We centre two funded co-produced, participatory and arts-informed projects that have been co-designed and co-led with disabled young people and people living with chronic (respiratory) illness. We critically explore some key processes, which we suggest can mitigate forms of disablism and ableism inherent to research processes which traditionally make them undemocratic spaces of inequity. Our paper offers original analyses into the very notion of democratic research which have significant applications; driven as they are by the presence of disability. These include (i) Crip time - the recognition of (disabled) people’s need for flexible forms of time; (ii) virtual methods and intimacies as routes to equity in research leadership; and (iii) flexible and slow/er research approaches. We also draw upon the ways in which the Covid-19 global pandemic has reshaped methodologies and approaches to inquiry. We advocate that, as research communities, we must come together to keep hold of these new inclusive and hybridised ways of relating and engaging in what are problematically framed as “post-Covid” times. We conclude by emphasising the importance of always committing to disrupting power dynamics through centring flexibility, accessibility and inclusivity across our inquiry with marginalised others.

## Introduction

In this article, we explore the power and potential of democratic research methodologies in and beyond Critical Disability Studies. Critical disability studies is an interdisciplinary community of researchers, activists and artists that not only centres disability but also considers disability as the driving subject of inquiry. All of us have experienced a sense of disciplinary dislocation at times when disability is ignored in our course reading lists, forgotten in our colleagues’ scholarship and research, and sidelined in discussions of equality, diversity and inclusion. Indeed, one question we would ask of the reader is; how often is disability the driving subject of conversations about democratic research? We suspect, with some annoyance, that disability is bolted on to debates rather than being part of the starting points of scholarship and research. Critical disability studies scholars seek to redress these omissions, those moments of neglect and times of ignorance. By centering disability we do so knowing that disability not only brings something to the table but, in many cases, disrupts and disturbs research (democratic or otherwise). And so throughout this article we will make ready use not only of the lessons we have learnt collaborating with disabled researchers and others, but also of some key concepts from critical disability studies.

In relation to our positionality as authors, then, we want to be transparent about our relationships with and to disability, and make visible our disability experiences in recognition that this has shaped the methodologies and approaches to research centred in this article. We have come together as disabled community co-researchers, experts-by-experience, artists, university researchers, carers of disabled people, and one of us is a clinician (a respiratory physician) working to support one of our projects. Thus, we span different kinds of engagements and experiences of disability and chronic illness. The majority of authors who have co-written this article have lived and embodied experience of disability and chronic illness, most living with life limiting and life threatening impairments (hereby LL/LTIs). Others of us are parents of disabled adults; some are lifelong allies to disabled people’s communities; and others are non-disabled researchers who have conducted sociological research in clinical and health contexts with marginalised and minoritised people.

For some of us, critical disability studies research and inquiry is underpinned by scholar activism; a form of advocacy and activism that ‘uses’ research contexts to challenge, trouble, dismantle and uncover the myriad oppressions and injustices that impact disabled people and their families. According to Runswick-Cole et al. (2022), scholar activists believe they have a role to play in creating social justice. Scholar activism positions research as a political endeavour and we follow emancipatory and participatory approaches to disability research, which have emerged alongside disabled people’s claims for civil rights (Oliver, 1992). Thus, from the outset disability research is a ‘democratising activity aligned to disability politics with ethical approaches rooted in social justice and equity’ (Liddiard et al., 2019: 154; see also Zarb, 1992). It’s important to note that, across the projects discussed in this article, scholar activism hasn’t subsisted as an individual act: but a form of collective action that centres collaboration, solidarity and a repositioning of disability as *the* driving subject of research and innovation. In short, disabled and chronically ill people, and their families and allies, have contributed as participants, co-researchers, and research leaders; charities, disabled people’s organisations (DPOs) and arts organisations have been and remain vital partners in the work; and our broader advocacy, artistic and activist networks have supported, encouraged, and disseminated findings. Moreover, when we conceive of the notion of scholar activism we firmly believe that the former (scholar) is enhanced by the aspirations of the latter (activist). This original insight contrasts with what we might call a lurking suspicion in academia: that activists do not make good scholars and that scholarship is watered down by activism. We contest this suspicious attitude and, instead, seek to illuminate the ways in which the political activism of researchers can drive critical intellectual work.

## The projects

To demarcate and unpack what is meant by making a commitment to democratic methodologies we centre two funded, co-produced, participatory and arts-informed interdisciplinary research projects - as case studies - that have been co-designed and co-led with disabled young people and people living with chronic illness. We offer these projects due to the ways they challenge/d the routine disablism and ableism that subsists in social sciences research. To define some key terms, ableism refers to a world view - a dominant grand narrative - that prioritises and values those deemed to be able-bodied and minded. Disablism is the resultant oppressive treatment experienced by disabled people and their families when they don’t meet such expectations. Where we refer specifically to ableism or disablism, we use the respective term. However, at times we use the term ‘dis/ableism’ in this article to refer to the dual processes of disablism and ableism. This is because, more often than not, ‘they work in conjunction, supporting one another, always intimately connected’ (Liddiard, 2018: 4).

### The Living Life to the Fullest Project

The Living Life to the Fullest project (ES/P001041/1) explored the lives of disabled young people living with what are known as ‘life limiting’ and ‘life-threatening’ impairments (LL/LTIs) and their families. These types of conditions are typically progressive and for many young people can mean short or shorter lifespans. Through its involvement with young people, the project quickly became a space that contested the culturally dominant discourses which position short/er lives as “lesser lives” in dis/ableist contexts in which ‘cultural responses to these young people are shaped by dominant discourses associated with lives lived well and long’ (Goodley et al., 2017: 197). Or as Garland-Thompson (2012: 351) argues, ‘…a life trajectory that is unpredictable or uncontrollable is anathema to our liberal modern ethic of self-determination, design, and freedom’. We found - through the stories of disabled young people with LL/LTIs and their families - that short/er lives, regardless of length and span, are vital, desired, and valued. In the Living Life to the Fullest project, collecting the stories of disabled young people with LL/LTIs through arts-informed and narrative methods enabled a disruption of dominant neoliberal-able (Goodley et al., 2017) fantasies surrounding lifespan, quality of life, and productivity as the root of human worth. The Living Life to the Fullest project had a focus on futurity embedded in an affirmative politics of disability – acknowledging disability as both valued and valuable: that which can bring new perspectives to a world obsessed with reifying normalcy and eradicating difference (see Goodley et al., 2021). The project was co-produced with and alongside disabled young people purposefully because ‘knowledge production about and around the lives of those with LL/LTIs rarely comes from disabled children and young people themselves’ (Liddiard et al., 2019: 1474). We centred virtual methods in our approaches. Virtual approaches are argued to be transformative within social and educational research (Hewson, 2014), and disability researchers have long emphasised online or virtual research environments as being of significant value to existing and emerging disability research methodologies (Carr, 2010; see; Liddiard, 2013).

### Cripping breath

Towards a new cultural politics of respiration (226472/Z/22/Z) is a 5-year Wellcome Discovery Award project that centres the lives of people who have had their lives saved and sustained by ventilatory medical technologies. In short, the original desire to think critically about ventilation emerged from the lead author’s own lived experiences of being and becoming a ventilator user as someone living with respiratory failure. In 2015, a routine sleep study revealed that her oxygen saturation was dropping over 30 times a night. A consultant handed the ventilator to her and told her to go home - “it’s easy to use” she said, “this is the on/off button.” In that life-changing moment, with no follow up, support, or acknowledgement of what this would mean for her life moving forward, let alone the emotionality of realising that one could no longer breathe independently, she was struck by an urge to learn about others’ experiences of living on ventilation. As a broader team, we also want to Crip dominant understandings of ventilation and ventilated lives: the idea that being on ventilation means one is near end-of-life; has a failing or ailing body; and ultimately, a life of lesser value (Abrams et al., 2021). With others, we also wanted to create a project which, through a mixed methods approach, could capture new understandings of ventilated lives: how ventilation is life-sustaining, enabling, relational, social, cultural - and fully embracing Crip - even joyful, pleasurable, playful.

Recently funded, the project brings together critical disability studies, medical sociology and humanities, science and technology studies, critical race studies and clinical practice to radically transform understandings of respiration and ventilation. In taking up the relative silences of breathing in and across these broad fields of study it asks: Where are the accounts and perspectives of ventilated people? Through provocative engagement with critical social theories Cripping Breath is forging new perspectives of respiration - an embodied, autonomous functionality constructed as central to our very humanness and the ability to live, the absence of which readily situates us at the very edges of death (see Solomon, 2020). The project’s transformative potential emerges, then, in centring ventilated people’s lived and embodied knowledge in existing theories of respiration and breathing for the first time. Cripping Breath will develop a new cultural politics of respiration in a time of new histories of ventilation emerging during an ongoing pandemic, and offers a Crip perspective - a sitpoint that emerges from disability studies and activism which unapologetically centres disability and chronic illness as valued human experiences. Across four distinct streams of inquiry - arts-informed, narrative, archival and ethnographic - Cripping Breath positions Crip perspectives as the very driving subjects of disability arts expression and curation, inclusive methodologies and scholarly transformation and equitable theory-building with, rather than on, marginalised communities. Like the Living Life to the Fullest project, Cripping Breath values disability and chronic illness for its productive, creative and disruptive potential, acknowledging the ways they offer ‘alternative ways of conceptualising the human subject’ (Braidotti, 2012: 37) currently omitted in existing scholarship surrounding breathing, respiration and ventilation.

In the remainder of this article, then, we centre these research projects as case studies to exemplify how they enact inclusive and democratic approaches to inquiry. In the first section, we briefly outline our understanding of Crip time and its value towards co-creating Crip perspectives, flexible and equitable methodologies. Next, we show how virtual methods and Crip time connect to enable authentic forms of project participation and leadership for disabled young people in research. We explore the relational benefits of virtual methods for research in and with disabled people’s communities. Later, we explore research inquiry that centres embodied knowledge and slow research and scholarship (Mountz et al., 2015), which we suggest has the potentiality to resist normative timescales of inquiry in the neoliberal academy. Throughout the article, we advocate the importance of committing to inclusive, accessible and flexible research design when co-producing and enacting democratic methodologies. In our conclusion, we emphasise that committing to such approaches means disrupting power dynamics as routine across our inquiry with marginalised and minoritised communities.

## The value of crip towards embracing crip time, perspectives and methodologies

Hamraie and Fritsch (2019: 2) define Crip as ‘the non-compliant, anti-assimilationist position that disability is a desirable part of the world.’ Crip purposefully pushes boundaries, works the edges, and contests normativity. This understanding follows others (see Clare, 1999; McRuer, 2006a; Sandahl, 2003) who have politically reclaimed and re-defined Crip from a dis/ableist slur to that which ‘questions – or takes a sledgehammer to – that which has been concretised’ (McRuer, 2006b: 35). Yet, Crip politics also position disability as productive, creative, vital and joyful. As we have written elsewhere (Liddiard, 2018: 38), ‘Crip shifts pathological discourses of disability that render bodies only as unintelligible and undesirable.’ Ultimately Crip is transgressive with its repositioning of disability and disabled lives as vital, valuable and dynamic (see Wilkerson, 2002).

Crip *time*, then, refers to the temporal relations of Crip, disability, and embodiment. Crip time is defined by Kafer, (2013) as the recognition of (disabled) people’s need for ‘flex time’ (see also Baril, 2016). Kafer, (2013: 27) elaborates that Crip time extends beyond needing *more* time: ‘It is this notion of flexibility (not just ‘extra’ time).’ For Kafer (2013: 27):

‘Crip time is flex time not just expanded but exploded; it requires reimagining our notions of what can and should happen on time, recognising how expectations of “how long things take” are based on very particular minds and bodies... Rather than bend disabled bodies and minds to meet the clock, crip time bends the clock to meet disabled bodies and minds.’

Thus Crip time is an affirmation that diversity in embodiment and barriers in the social world means life can take place across different timescales (see also Kuppers, 2014). Crip time is therefore a political acknowledgement that contexts of dis/ableism propagate timescales and temporalities that benefit non-impaired bodies and minds. Disability theorist Garland-Thompson calls this ‘normate’ time. Kuppers (2014: np.) articulates this specifically in relation to disabled embodiment: ‘...there is the day we lie in bed, the time of pain blooming in our bones, the end of the street impossibly far for limping legs, the meeting and its noise assault set against the reassuring tick of the wall clock at home’. Chazan (2023: 1) defines Crip time as ‘the non-linear, unpredictable, ever-changing, or multiply enfolded temporalities of being disabled’. For Rodgers et al. (2023: 1482), speaking in relation to how disabled academics manage the ableist temporalities in the neoliberal university, ‘the concept of crip and cripping time in relation to disabled academics opens up new ways of thinking, doing, and being that are not constrained by normative (clock) time that marginalises disabled subjects. White (2022: 5) argues that Crip time is ‘...a flexibility and an expansion of time, both in response to bodily necessity and to societal barriers that make it so that more time may in fact be necessary.’

In the following section, we use Crip time as an analytical tool. We explore how disabled and diverse embodiments in the Living Life to the Fullest Project infused normative research processes - which are understood as linear, charted, fast and which take place on ableist timescales - caused us to centre virtual methods and intimacies in research which we consider forms of Crip solidarity.

## “I do the majority of my work from my bed”: Virtual methods and intimacies as crip routes to equity in research leadership

The quote above was spoken by a disabled young activist, advocate and community researcher, Lucy Watts MBE. Lucy was Lead Co-researcher on the Living Life to the Fullest project. Prior to meeting Lucy, we had been struggling to mobilise a co-production approach which centred on a Co-researcher Collective of disabled young people. We had networked, put out calls, and gone to visit special educational need and disability (SEND) schools giving introductory research workshops in the hope of recruiting disabled young co-researchers to join the project. None of these had been particularly fruitful. At that time, Lucy was living with a number of life-limiting and life-threatening impairments (LL/LTIs); much of her advocacy was around young people’s palliative care and end-of-life planning, both of which she was experiencing in her personal life. “I do the majority of my work from my bed” was Lucy’s direction that we needed to find more accessible ways for disabled young people with a variety of embodiments and care arrangements to become co-researchers in the project. It also emphasised that her labour, across multiple organisations, was deeply valuable and valued, *because* of her lived experience (which included long periods of hospitalisation and bedrest), not in spite of it. On reflection, it’s possibly one of the most powerful descriptions of Crip time: that quite literally working from your bed in capitalist cultures is already deeply radical practice. As she always did, Lucy gave her (precious) time to us and became Lead Co-researcher on the project. From these conversations came a revised approach whereby our Co-researcher Collective took place predominantly only through virtual means and methods. We made sure that our co-researchers had everyday roles and responsibilities in relation to the project and we worked together to collaborate on every stage of the project (see Liddiard et al., 2019). We outline these below and suggest that doing this only through virtual approaches offered a necessary cripping of time which enacted flexibility, accessibility and accountability.

Importantly, Lucy used her vast networks to recruit co-researchers; together we worked on advertisements, which she would share with her networks and communities. Her existing roles, for example, as ambassador for the national children’s palliative care charity, *Together for Short Lives* and being a member of the NHS Assembly, as well as a host of other roles (e.g. volunteer, patron or trustee) for disabled young people’s and palliative care organisations were key to this process. Lucy has also led research into sexuality and disability as part of the Sexuality Alliance (Blackburn et al., 2019). Lucy was also very well known nationally through her TEDx NHS talk in which she discussed her own experiences of palliative care (Watts, 2019), and had received an MBE for her services to disabled young people in the Queen’s New Year’s Honours List 2016. Thus, she served - at this stage - as a ‘community connector’, people who Wallace et al. (2019: 366) state ‘undertake valuable boundary work within the community to include people who are hardly reached.’ It’s worth noting that disabled young people living with life limiting and life threatening impairments (LL/LTIs) are typically excluded from even disability research, being considered ‘hard to reach’ (Liddiard et al., 2022), and so Lucy’s networks were vital. Within a matter of weeks, Lucy had recruited a vibrant group of disabled young women living with life-limiting and life-threatening impairments (LL/LTIs) to the Co-researcher Collective and taught us (pre-Covid) about the value of virtual methods towards creative and flexible research design, and its importance when researching disabled young people’s everyday life worlds.

Through virtual research environments, the Co-researcher Collective was able to actively and meaningfully co-lead inquiry, which included: ‘(i) supporting research design through discussion (planning both narrative and arts-informed approaches in the project); (ii) co-writing interview schedules for young people and parent participants; (iii) recruiting participants for data collection and carrying out online interviews through email, Facebook Messenger and Skype; (iv) planning the project’s impact strategy and building relationships with impact partner organisations; (v) working with our community research partner organisations; (vi) meeting regularly via the Research Management Team to co-manage the research process as a whole; (vii) writing blogs and making films that communicate and document our processes and preliminary findings; (viii) presenting at conferences and research festivals; (ix) undertaking various public engagement and knowledge translation activities (online and offline); and (x) co-authoring articles for publication’ (Liddiard et al., 2019: 157). This labour shows the extent to which disabled young people were located in the project as co-leaders, having access to forms of everyday leadership and opportunity typically denied to disabled young people living with life-limiting and life-threatening impairments (LL/LTIs) (Abbott and Carpenter, 2014).

When we say ‘virtual methods and environments’ we think it’s important to be clear as to our practice: we used Whatsapp for ‘speedy’ and responsive project communication (we discuss Whatsapp later in regards to friendship and intimacy); Google docs for co-authoring to be able to write together in ‘live’ time; Google Calendar and Google Meet for project organisation and meetings; and Facebook Messenger, Skype, and email for data collection and collaborative analysis. In this way, we created our own ‘virtual world’ within the project, whereby we were able to co-lead in accessible ways together. This virtuality enabled us to *flex* time, creating Crip-friendly forms of research inquiry, as we have reflected upon elsewhere (Liddiard et al., 2019: 163):

‘For example, co-researchers will often message us at all times of the day and night; we schedule meetings around the presence and time of care visits and support from personal assistants; Skype meetings involve breaks to adjust tracheostomy tubes or seat cushions; blog posts and tweets get written during the night; online interviews via Facebook Messenger are meticulously broken down into multiple sessions due to exhaustion on behalf of the interviewee and/or the co-researcher; contributions require regular breaks due to frequent hospitalisations and planning a ‘physical get-together’ (e.g. to a conference) can take considerable time and labour due to the need to manage multiple barriers to access. We do not mention these here as negative impacts of impairment, but as vital moments to rethink and reconsider conventional temporalities of qualitative methods and research processes.’

We suggest that co-creating this Crip approach also worked to the benefits of our participants - disabled young people with LL/LTIs and their families - ensuring that the materialities of disabled body-minds are centred in inquiry, rather than written out and overlooked. Moreover, it enabled our disabled young co-researchers to contribute to inquiry in meaningful ways, not only challenging the tokenism that can plague research with research with children and young people (see Coad and Lewis, 2004) and disabled people (Liddiard, 2013), but creating forms of work that offered different kinds of remuneration. We established a budget to fund co-researchers to purchase technology of their choice as recognition of their commitment and labour within the project. We also invited co-researchers to become members of the research centre the Institute for the Study of the Human (iHuman) at the University of Sheffield in acknowledgement that as researchers they should have access to research communities and offered co-researchers university certificates and references for jobs, education and scholarship applications as evidence of their contribution of expertise, skills and knowledge to the project. In related project work that followed, we paid co-researchers an hourly standard postdoctoral pay grade in recognition that giving co-researchers a budget for technology was not enough to recompense their contributions.

## Building crip alliances and solidarity: emotionally engaging in inquiry

Not only did our engagement, and later reliance (as the Covid-19 pandemic then hit), on virtual research environments support an inclusive, radical and democratised research process that took place in Crip time shared with disabled young people, it created unexpected intimacies - friendships and relationships which became equally valuable to collaborative inquiry. To give an example, the Co-researcher Collective co-led the analysis of artistic and narrative data in the project, supported by a hybrid residential ‘Analysis Retreat’ hosted in an accessible hotel over 3 days. The aim of the Analysis Retreat was for co-researchers to come together both in person and online to collaborate on ‘immersing ourselves in participants’ stories and sharing our own experiences as part of the analytical process’ (Liddiard et al., 2022: 6). Emphasising the extent of our virtual approach, and centring of Crip time, the Analysis Retreat was one of the only times that (some) co-researchers met in person in a 3-years project. Despite this geographical and physical ‘separation,’ our relationships across the project were highly intimate: exploring disabled young people’s experiences of what it means to live short/er lives both necessitated and encouraged an emotionality and reflexivity in the research. Within the project we readily positioned co-researchers as ‘theoretical provocateurs and theorists in their own right who, through their activism and writing, are challenging us to reconsider the meaning of life, death and disability’ (Liddiard et al., 2019: 1473). In our project text, co-authored with co-researchers, we reflected on the affective meanings of the Analysis Retreat (Liddiard et al., 2022: 6):

‘This was a very impactful time for those involved and we believe that being together in a safe space allowed a greater emotionality into the process as we examined our own lives. In fact, each one of us shed a tear during this time. We feel the mutual understanding and friendships that have developed through our meetings and WhatsApp conversations have led to increased intimacies, facilitating a richer content for this book. The Co-researcher Collective became a force of its own and our relationships became deeper than a team simply collecting and analysing data together. We have both commiserated and celebrated with each other through life events from moving locations and struggling to recruit personal assistants (PAs) to finally being successful in winning ‘fights’ for [social care] funding. Throughout, there has been an innate understanding of the challenges these things pose and it has become a safe space to voice these frustrations within the group where so often these experiences are silenced’.

Moreover, co-researchers explained that their emotional ties to one another were enabled through an understandable solidarity, as well as ‘access intimacy’. Disabled feminist activist Mingus (2011: np.) coined the term access intimacy to refer to that ‘elusive, hard to describe feeling when someone else “gets” your access needs. The kind of eerie comfort that your disabled self feels with someone on a purely access level.’ The Co-researcher Collective reflected (Liddiard et al., 2022: 7):

‘This access intimacy that we enact together as a team denotes closeness, friendship and solidarity in our project as ways to extend thinking about the affective politics and emotionality of inquiry. We note this here, because despite these intimacies, in nearly four years of working closely together, we have seldom ever been in the same room, or shared physical space. Our point here, then, is to counter normative ideas of face-to-face work as a point of superiority in qualitative research and to affirm technologies as spaces ripe for human and affective connection, nurture and care, especially for marginalised people who experience barriers in the physical and social world.’

For all co-researchers, then, the Living Life to the Fullest project was experienced as an emancipatory process (Oliver, 1992). Emancipatory research refers to ‘a radical new approach to researching disability’ (Barnes and Sheldon, 2007: 234) that challenges the typical power imbalances in much social scientific research into disability. Grounding the social model of disability, it advocates giving control of research processes to disabled people, ‘to make disability research more relevant to the lives of disabled people’ (Oliver, 1992: 109). As Barnes (1992: 122) states, emancipatory research is primarily about ‘the systematic demystification of the structures and processes which create disability and the establishment of a workable dialogue between the research community and disabled people in order to facilitate the latter’s empowerment.’ Thus, a research space which advocated co-researcher power, control and leadership led to an emancipation through the process. Thus, key legacies of the project not only included strong, continuing friendships, but employment opportunities, access to further and higher education, and further research leadership. These material outcomes in the lives of disabled young people living with life-limiting and life-threatening impairments (LL/LTIs) cannot be underestimated: this is a category of disabled young people who experience high rates of education exclusion and unemployment, and all of the subsequent material precarities that come with such structural marginalisation. More than this, though, is that *access* to the research process, to analysis, theory and writing (Whitney et al., 2019), and to experiences of leadership, meant the project was a space of self-reflection and revelation; an affirmative space for the growth of confidence, esteem and self-worth in dis/ableist contexts, again where this is readily denied for disabled young people (Liddiard et al., 2022: 7):

‘Joining The Co-Researcher Collective has benefitted me [Sally] personally in addition to being a group doing fab quality research into the lives of young, disabled people. This is because it is a group formed of amazing, strong, young disabled women who are making waves in and outside the spheres of disability studies and activism. They have challenged me to believe in myself more, value and trust my own experience and allowed me the space to grow in the field of research. Not only that but they are bold, powerful women who have taught me that I am far more capable than we realise and inspired me to push the limits of my own and society’s expectations.’

Thus, our research relationships with one another in the Living Life to the Fullest project were intimately connected to our egalitarian research politics: we actively worked towards generating a politicised and transformative research environment that acknowledged and valued the lived, embodied and affective realities of young people with LL/LTIs. We now turn to further unpack ways of valuing marginality, embodiment and accessibility as key to democratising research processes, showing the ways that alternative temporalities of research are beneficial to interdisciplinary co-produced and participatory research.

## Taking a breath: thinking about crip time and slow scholarship in universities in a pandemic age

Like the Living Life to the Fullest Project, Cripping Breath actively locates lived and embodied experience at the very heart of inquiry. Cripping Breath Crips time by embracing ‘slow scholarship.’ It does so to push the boundaries of what’s possible (or not) in the neoliberal academy to play with the temporalities of normative research processes which are typically fast-paced, metric and output-oriented, inaccessible to many (and thus exclusionary), and which are fixed to accelerated timelines and follow the temporal regimes of the neoliberal university. Slow scholarship ‘questions the ever-increasing demands of academic life, placing them broadly within wider tendencies toward neoliberal university governance’ (Mountz et al., 2015: 1238). It involves resistance, engaging slowly with the object of study, engaging with others and improving the quality of academic practices such as writing (Mounts et al., 2015). Cripping Breath is spread over 5 years, which was purposeful towards acknowledging that the relational elements of co-production take time (as we learnt in the Living Life to the Fullest project, and from Lucy); that relational labours and a feminist ethic of care involve relational skills, such as ‘empathy, reflection, anticipation, affirmation and compassion’ (Katzman et al., 2020: 519), and that these must be slow/er. We also needed different approaches to time because our Research Team comes with various types of impairment, embodiment, and chronic illness (many of us live on ventilation) which can mean regular minor illness, hospitalisation, fatigue and breathlessness. As such, those typically excluded from research processes and knowledge production can take centre stage (see Whitney et al., 2019).

Furthermore, co-leading a project about respiratory illness in a pandemic age involves a consideration of danger and a desire to encourage safe spaces which work to mitigate the routine forms of risk disabled people (and others) now live in during a “post-Covid” time where many forms of risk management in the UK (e.g. CO_2_ monitors, masking, free access to Covid-19 testing) are considered unnecessary. Covid-19 remains dangerous to many of the Research Team (see Liddiard et al., 2021). Like Johnson et al. (2024: 211, we also acknowledge that the ‘COVID-19 pandemic brought forth multiple and at times conflicting temporalities that reordered our sense of time.’ They explain that, for some, Covid-19 caused a ‘speeding up’ of time, the necessity to adapt quickly to new ways of being, working, and relating to others. Yet for some, Covid-19 - and particularly lockdowns - brought a *pause* to life - a halting and slowing down of different facets of life. Johnson et al. (2024: 211) rightly argue for greater consciousness towards the ableism and disablism inherent in the rush and speed to “return to normal”:

‘We observe, with puzzlement and concern, how desires to return to normal are accompanied by shrinking access to testing, removal of masking mandates, lifting of gathering limits and other quarantine requirements, and reduced opportunities for remote access.’

They further note that this reduction of protection, for disabled people and others, enacts a ‘necropolitical logic that devalues disabled life by unevenly exposing certain populations to the possibility of death’ (Johnson et al., 2024: 211). In Cripping Breath, then, we will resist these necropolitical impulses via normalising a hybrid approach across the project, actively pushing back the desire to “return to normal” in research contexts, which is particularly prevalent in university cultures where presenteeism - ‘exhibiting excellent attendance and working elevated hours’ (Hadjisolomou et al., 2022: 570) - is creating precarious conditions for all workers during a continuing pandemic. Thus, we have a Crip desire to develop inclusive and Covid-safe research practices and processes for all, but particularly for our participants, collaborators and Research Team members who have been situated in Clinically Extremely Vulnerable (CEV) categories in relation to Covid-19. Our approaches ensure a responsive and safe(r) project that is flexible in the face of a continuing global pandemic and which enacts inclusive and positive research environments to include people with myriad clinical vulnerabilities.

More broadly, creating a Covid-safe and accessible research environment can, and should, take time. This means that Cripping Breath will flex normative timescales of research; for example, data collection. Communicating all public-facing research materials in Easy Read, British Sign Language (BSL), plain English and accessible animation; writing and enacting Covid-safety policies for participants and others; ensuring personal assistance support at all participant workshops, meetings, and research events; undertaking multiple and complicated ethics applications and processes; embedding risk assessment throughout the process as routine; Cripping recruitment processes to rethink institutional constraints around employing research associates and remunerating co-researchers; and working with diverse embodiments means Cripping Breath is, very purposely, a slower (research) process. As a co-produced project, we also know that, in general, co-production is an approach to inquiry that requires an adaptability with regards to time.

A project that centres the lives of people who live on or with ventilation also means considering the role of death within the project. This consideration has been further affirmed in the Cripping Breath Research Team, as members voiced recently losing colleagues and friends in and across their research, artistic and advocacy work. In the Living Life to the Fullest project, we had to manage the deaths of participants, which was incredibly hard; and in 2023 Lucy died, which painfully rippled through the team in ways that are hard to write here. Thus, death in inquiry exploring the life worlds of people living with life-limiting and life-threatening impairments necessitates an embracing of both death and loss. We’ve no doubt that death, dying, fragility and grief will likely be ever-present themes within Cripping Breath. Negotiating these - both practically and emotionally - means *bending* time within the research process in a number of ways. Making space for bereavement and emotion as routine; hospitalisations, recovery, illness and decline curving project activities; while death halts everything - and the grief after loss transcends through the project in myriad ways stretching and tightening as it goes.

## Co-creating institutional change: cripping project structures

Moving forward, much of Cripping Breath, as a bid for funding, was developed collaboratively and virtually, embedding very similar approaches to team working as in the Living Life to the Fullest project. Importantly, the lead author obtained internal funding to bring a disparate research team to come together and co-design the project and co-author and submit our initial bid for funding, as well as co-produce preparation materials for our interview with the funder. All team members were paid for their part in this process, acknowledging that ethical forms of co-production begin way before a project commences (see Liddiard et al., 2019). Furthermore, a key aim of Cripping Breath is to develop the Crip politics and methodologies we instigated in the Living Life to the Fullest Project, but use these to aim for institutional changes to encourage more inclusive research cultures and working practices in our universities. For example, thus far in Cripping Breath, which has only just formally begun, our researcher posts in the project will not necessarily require doctoral experience or study, resisting academic desire for qualification within research roles, and these posts will be fully remote if required, to push back at presenteeism as an ableist underpinning of what work looks like in a university context (see Magalhães et al., 2022).

This has led us to critically explore inclusive forms of recruitment: removing ableist language from job descriptions and person specifications; flexible forms of interview; accessible inductions and onboarding; and flexible and remote working as routine. Furthermore, Sally Whitney-Mitchell, a co-researcher from the Living Life to the Fullest project has been formally employed by the University in her new role in Cripping Breath as Co-researcher Lead, and our aim is for all co-researchers to be employed on fixed term contracts, giving them access to employment rights, opportunities and career development. This has meant a lot of advocacy and bureaucracy by the lead author in her role as principal investigator (PI). For example, such flexible approaches to employment meant working closely with the university’s human resources (HR) teams because each individual co-researcher and Artist-in-Residence role had to go through the IR35 process - a review process to determine the legal and taxation terms between an individual and the university, instigated by off-payroll working rules (HM Revenue and Customs, 2019). We also have had to work closely with university finance teams to encourage learning about flexible forms of payment for our co-researchers, to ensure that payment does not create further precarity for those who receive income from state benefits. And we’ve worked with university contracts teams to ensure non-standard Collaborator Agreements, which build in flexibility and security for our partners (small disability and arts organisations), as well as promise co-ownership of data, findings and outputs. All of these acts and forms of ‘extra’ labour are designed to further democratise the research contexts and institutional environments in which we are working. All of these commitments to *change* relate to and connect with the need to bend time to instigate new temporal relations in Cripping Breath. Thus, critical disability studies research requires more than just flexible environments, but a total reimagining of scholarship and inquiry, and the institutional contexts funded research often sits within, according to Crip time as that which ‘bends the clock to meet disabled bodies and minds’ (Kafer, 2013: 27)

## Conclusion

We want to conclude this article, then, by emphasising the importance of always committing to disrupting power dynamics through centring flexibility, accessibility and inclusivity across our inquiry with marginalised and minoritised communities. We advocate that, as academics, researchers, artists and advocates - diverse research communities - we must come together to keep hold of inclusive ways of relating and engaging, particularly when our collaborations are situated within institutional contexts which demand particular temporalities and cultures in relation to bodies and labour. This is especially important for disabled researchers and/or critical disability studies research and other forms of participatory practice. Through our methodological reflections, we have also, we hope, encouraged social and education researchers and others to take up virtual environments and relationships when researching with marginalised and minoritised people when undertaking empirical explorations of their lives. We’ve shown how the virtual, digital and accessible connect to the intimate, affective, political and the temporal. By centering disability, we have revealed its productive potential, how it can disrupt and disturb research (democratic or otherwise) and offer new perspectives on democratic research approaches.
